# ChemProt-3.0: a global chemical biology diseases mapping

**DOI:** 10.1093/database/bav123

**Published:** 2016-02-14

**Authors:** Jens Kringelum, Sonny Kim Kjaerulff, Søren Brunak, Ole Lund, Tudor I. Oprea, Olivier Taboureau

**Affiliations:** ^1^Department of Systems Biology, Center for Biological Sequence Analysis, buildin 208, kemitorvet, Technical University of Denmark, DK-2800 Lyngby, Denmark; ^2^Department of Disease Systems Biology, Faculty of Health and Medical Sciences, Novo Nordisk Foundation, Center for Protein Research, University of Copenhagen, Blegdamsvej 3A, DK-2200, Copenhagen, Denmark; ^3^Translational Informatics Division, Department of Internal Medicine, University of New Mexico Health Sciences Center, MSC09 5025, Albuquerque 87181, New Mexico, United States of America; ^4^INSERM, UMRS-973, MTi, Universite Paris Diderot, 35 rue Helene Brion, 75205 Paris Cedex 13, Sorbonne Paris Cite, France

## Abstract

ChemProt is a publicly available compilation of chemical-protein-disease annotation resources that enables the study of systems pharmacology for a small molecule across multiple layers of complexity from molecular to clinical levels. In this third version, ChemProt has been updated to more than 1.7 million compounds with 7.8 million bioactivity measurements for 19 504 proteins. Here, we report the implementation of global pharmacological heatmap, supporting a user-friendly navigation of chemogenomics space. This facilitates the visualization and selection of chemicals that share similar structural properties. In addition, the user has the possibility to search by compound, target, pathway, disease and clinical effect. Genetic variations associated to target proteins were integrated, making it possible to plan pharmacogenetic studies and to suggest human response variability to drug. Finally, Quantitative Structure–Activity Relationship models for 850 proteins having sufficient data were implemented, enabling secondary pharmacological profiling predictions from molecular structure.

**Database URL**: http://potentia.cbs.dtu.dk/ChemProt/

## Introduction

Many chemical biology initiatives in Europe and the USA aim to screen large compound collections with dedicated bioassays i.e. EU Lead Factory (1), EU-Openscreen (2) or BARD in the USA (3). Such large initiatives generate large amounts of data that support academic and industrial research in the discovery of safer chemicals, with better efficacy. To make chemical biology information accessible to scientists, several repositories of bioactive small molecules have been developed: ChEMBL (4), PubChem (5), ChemSpider (6) and OpenPhacts (7) are the largest, more general databases available to the public. The National Institutes of Health’s Molecular Libraries Program (MLP) funding developed the BioAssay Research Database (BARD), focusing on assay ontologies for PubChem bioassays (3).

Advances in chemical biology and systems biology have shown that most drugs interact with multiple targets and that the pharmacological profile of a drug is not as reductionist as once believed (8). Moreover, proteins rarely operate in isolation within and outside cells but function in interconnected pathways instead. Given the integration afforded by systems biology, it is now possible to consider a more general physiological environment for protein targets and biological processes. As massive amounts of data are generated and accumulated via new experimental technologies such as transcriptomic, proteomics and genomics (through next-generation sequencing), drug action can be explored across multiple scale of complexity, from molecular and cellular to tissue and organism levels (9–11).

Multi-target pharmacology exploration increases when information linking the relationship between chemical and target spaces is readily available. As archived data are processed and homogenized, our total knowledge on protein−ligand interactions is increasing at an amazing pace (12, 13). Scientists having access to these data, approaches such as chemogenomics, proteochemometrics and polypharmacology have started to emerge (14, 15). These help to mine evaluate and ultimately distil this vast amount of protein–ligand interactions data, enabling the predictions of single ligands against a set of heterogeneous targets (16).

This third version of ChemProt is not a simple update for disease chemical biology data. Rather, we provide a friendly platform to navigate through the various data sources, from global evaluations to a focused analysis. Several computational approaches are included: ligand-based similarity, target-based promiscuity, QSAR (Quantitative Structure–Activity Relationship) methodology and network biology-based enrichment analyses. These approaches support novel hypotheses generation for bioactivity of novel and already-annotated compounds, and the ability to identify additional genes that may play major roles in modulating chemical perturbations in man. The updates and new methods introduced in ChemProt-3.0 are presented below.

## Data sources

We updated all the chemical protein interactions data from the open source databases ChEMBL (version 19) (4), BindingDB (17), PDSP Ki database (18), DrugBank (version 4) (19), PharmGKB (20), IUPHAR-DB database (21) and STITCH (version 4) (22). Clinical information from the Anatomical Therapeutic Chemical Classification System (23) developed by the World Health Organization, as well as side effect data from Sider 2 were also integrated (24).

From a biological perspective, we updated our internal human interactome platform to reach 14 421 genes interacting through 507 142 unique PPIs (25). OMIM (26), the human disease network (27) GeneCards (28), KEGG (29), Reactome (30), UniPathway (31) and Gene Ontology (32) databases were also downloaded, curated and included in our system. Overall, the integrated data sources were increased by over 60% compared to the earlier version.

As many different data types were aggregated in ChemProt, a ‘zChemProt’ value for each compound-bioactivity interactions was computed for visualization in the several heatmaps developed. Basically, for each of the 11 most prevalent data types (IC_50_, EC_50_, Potency, AC_50_, pIC_50_, Log K_i_, pK_i_, pEC_50_, *K*_d_, *K*_i_), a zChemProt value was computed using the mean and standard deviation calculated from the distribution of the associated data types for each target in a similar way described in CARLSBAD database (33). IC_50_, EC_50_, Potency, AC_50_, *K*_d_ and *K*_i_ were log transformed before computing the zChemProt values. Large values indicate strong chemical–protein interactions and are represented in orange. Low value (weak interactions) is depicted in blue.

## Predictions methods

Daylight-like 1024 bit fingerprints was computed with RDkit (www.rdkit.org) and the chemical similarity between two compounds was quantitatively assessed using the Tanimoto coefficient (34). The Similarity Ensemble Approach (SEA) (35) has been re-compiled on the ChemProt server and updated according to the novel zChemProt data and integrated into ChemProt 3.0. Only proteins with >10 chemicals were included for SEA prediction, using the same protocol as described in the previous version (36). For sequence analyses, protein sequences were obtained from Uniprot (37). Sequences comparisons were computed using BLASTP and estimated to be similar when their *E* value was lower than 10^−^^10^ (38). All compounds were decomposed into ring scaffolds based on an internal implementation of the ‘Scaffold Hunter’ hierarchical classification algorithm (39–40) with the addition of decomposition of non-ring molecule based on rules 7–10, as described by Schuffenhauer *et al.* (41). This hierarchical decomposition allows the generation of scaffold trees enabling an easy and interactive navigation of the chemical biology space in large datasets and the identification of potential new compound classes with desired bioactivity.

For this release, QSAR models were trained for each protein with >20 chemicals (in total 850 proteins). A Naïve Bayes classifier was trained using 5-fold cross-validation for performance assessment. Features selection, five different computational fingerprints (Daylight and Morgan fingerprints) and three different cutoffs (−log 10 value: 4, 5 and 6) for classifying active and non-active compounds, were used to produce overall 15 classification models for each target. To predict new compound, each model was weighted by the cross-validated performance measure resulting in a prediction value between 0 and 1 (where 1 is high predicted binding). To avoid bias toward negative or positive data, each of the three datasets used for training were balanced by including as many negative compounds as positive including random compounds from the ChemProt database. The performance of the developed QSAR ensemble approach was tested on a dataset of hERG binders and showed an improved performance compared to a previous reported study (42) (Aroc = 0.827, Matthews Correlation Coefficient (MCC) = 0.488 using 5-fold cross-validation). Furthermore, the method was benchmarked against the SEA implementation on a dataset consisting of 143 proteins with associated activity values from the ChemProt-3.0 dataset. In a 5-fold cross-validation scheme on each of the 143 proteins, the QSAR models outperform SEA (*P_val_* = 2.2e *^−^* *^16^*, paired *t*-test of Spearman correlation coefficients for each protein predictions). However, models developed from limited amounts of data might not provide reliable predictions. The user can consult the ‘prediction info’ tab (by clicking on the protein of interest on the heatmap) to obtain the number of molecules used for training the QSAR models. Details about the procedure are described in Supplementary Information.

## Visual interface

**Figure 1.**
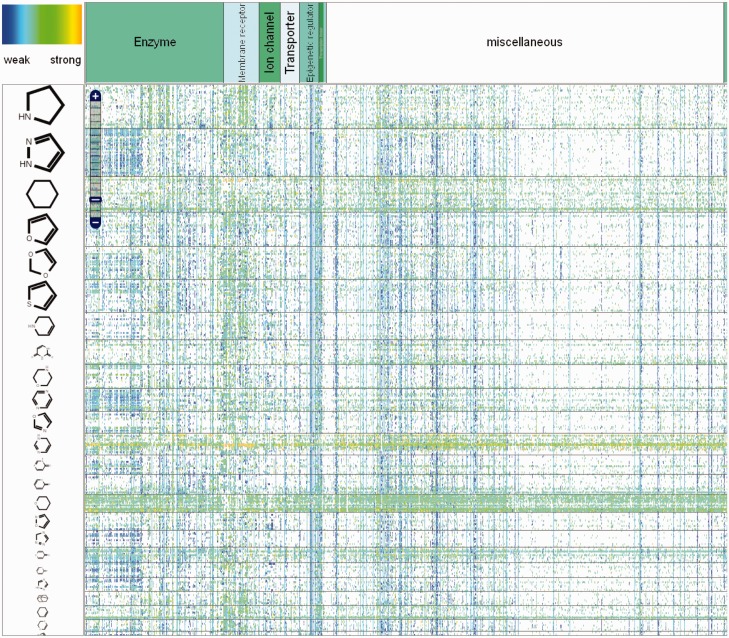
Global view of the chemical-protein interactions heatmap in ChemProt.

In ChemProt 3.0, the front page was modified to have all the functionality available on the page. The user has the possibility to search information about a compound, a protein and a clinical outcome, or he can choose to perform a QSAR prediction for a specific compound. A molecule can be imported as a SMILES code, or alternatively it can be drawn or uploaded from a compound structure file via the SD file format. A new function called ‘Heatmap’ was integrated, which allows the user to have a global view of chemical-protein interactions (Figure 1). In this graphical interface, the user has the possibility to localize the bioactivity associated to a requested compound, a set of defined compounds or a set of similar compounds based on the chemical structure. Several layers of granularity have been implemented on the heatmap. The proteins have been categorizes by families, using the protein classification tree implemented in ChEMBL and the compounds have been decomposed in scaffold and chemical groups based on the scaffold tree implementation similar to CARLSBAD. This gives the user the opportunity to visualize scaffold-protein activity relationships. A color spectrum from blue (low activity) to orange (strong activity) is used to indicate the activity. All compounds structures and protein IDs (based on Uniprot) are clickable, which gives access to more detailed information about physicochemical properties and protein function respectively.

An interesting feature with this graphical interface is the possibility to match other biological to chemical data. Instead of choosing ‘for drugs’, the user can select targets, pathways, diseases or side effects and see the association between chemicals and these endpoints.

From the protein ID, the user has access to the proteins complex (represented as a protein’s network from protein–protein interaction data). The complex of proteins is then mapped to biological terms such as diseases, GO terms and pathways with a corrected *P* value to evaluate the significance of these associations.

Finally, the user has the possibility to download the results in flat-file format to perform others analyses.

Methodology: Daylight-like fingerprints, defined by 1024 fragments were computed using RDkit (www.rdkit.org). The QSAR models were trained using scikit-learn software (http://scikit-learn.org/stable/). The visual interface was implemented using HTML 5 and JavaScript. The webserver is limited for an input file of 50 molecules (in SMILES or sdf file) per query to limit the time necessary to get the output. For larger queries, the user is advised to contact us.

## Applications

**Figure 2.**
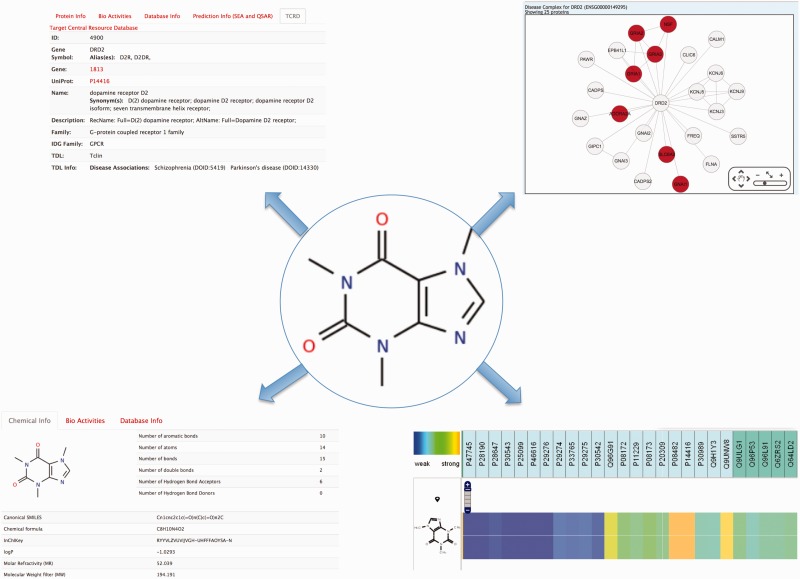
Information that can be collected from a search on caffeine. Top left, functional information on bioactive proteins for the query compound is depicted. Bottom left, chemical and physicochemical information is gathered. Top right, protein’s complex associated to the chemical is shown and the bottom right is depicted the protein’s annotation and prediction (through QSAR) for caffeine.

Caffeine, a well-known natural product extracted from coffee beans or tea leaves, is often used as a central nervous system stimulant (43). Several outputs can be displayed like those shown in Figure 2. Typing the compound name in the ‘Compound’ field and clicking on the Submit button, the user is redirected to the global chemical-protein heatmap with the query compound showing up in the compound list as default. Any compound can be added to the list by writing a new compound name in the ‘search’ box. Clicking on the ‘flag’ 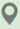 next to a compound name in the compound list prompts the heatmap to zoom in or out from that specific compound, enabling a fast way to visualize the proteins signature for the queried compound, as well as for compounds sharing scaffold similarities. Clicking on the ‘fingerprint’ logo 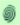 in the vicinity of the compound name, a chemical structure similarity profiling can be performed, enabling the user to visualize and to navigate within that pharmacological heatmap. For the Caffeine example, 105 similar compounds (with a Tanimoto coefficient > 0.85) were found, with bioactivities associated to 449 proteins (from weak in blue to strong in orange). The user is able to zoom in the heatmap and to narrow the information from the classification proteins tree to specific proteins (defined by uniprot ID). The user has also the possibility to navigate inside the heatmap. One option is to fill out missing values by choosing ‘SEA’ or ‘QSAR’ under prediction in the top of the page.

By clicking on the compound structure, physicochemical features [such as the Lipinski rules (44)], number of proteins with bioactivities and the databases from which the information was gathered, are shown. Similarly, the user can click on a specific protein name and get more information on the function of the protein, diseases associated to this protein and predictions based on SEA and QSAR. For example, under ‘family A GPCR’, caffeine is shown to be potent (35.5 nM) on the rat muscarinic M1 acetylcholine receptor (P08482). It also shows a strong association with the dopamine D2 receptor (P14416) based on the STITCH system. By clicking on this protein, the user is presented with information on this dopamine receptor. Notably, disease associations are queried through the TCRD (Target Central Ressource Database Application: http://juniper.health.unm.edu/tcrd/) database and the genetic variation through the Ensembl database (45). A complex disease network is also associated to this protein. Clicking on this link, diseases (such as schizophrenia and epilepsy) and GO terms (plasma membrane, ligand-gated ion channel activity, etc.) are shown.

Instead of looking for ‘functional’ protein annotation, it is further possible to select ‘pathway’ or ‘diseases’ for caffeine (also for the set of 105 similar compounds). The heatmap will be depicted according to the query. Each protein annotation (functional, pathway, disease) is presented in a tree format. Proteins have been categorized from families to proteins using the protein target tree implemented in ChEMBL. It has been done similarly for the pathway using the unipathway (31) implementation tree and disease using the human disease network (27). For example, using the disease heatmap, strong associations are found between caffeine and ventricular tachycardia, slow acetylation, glycogen storage disease, dystonia and thyroid carcinoma.

Finally, from the ChemProt-3 front page, the user can write the caffeine’s SMILES in the QSAR prediction box, click on ‘Submit QSAR’ and then get a prediction of positive and negative bioactivities for the ensemble of proteins in ChemProt where reliable QSAR models can be produced. This option allows the user to have a direct QSAR prediction for a new compound not present in the ChemProt database.

## Conclusion

Given that access to many chemogenomics databases is possible, linking them to biological resources and using a number of machine learning tools, scientists can now estimate the bioactivity profile of molecules across a large number of targets, pathways, diseases and other clinical outcomes using ligand-based, target-based and network-based models. Such multi-target, multi-layer strategies are becoming more and more accepted by the scientific community. Within ChemProt, it is possible to navigate the chemogenomics space and to link chemically induced target perturbations to diseases and other biological outcomes. Such tools might be of interest for drug discovery, drug safety and also chemical risk assessment. ChemProt 3.0 supports predicting bioactivities on targets and off-targets for new compounds and can assist in the associations to phenotypes and side effects relationships.

## Supplementary Data

Supplementary data are available at *Database* Online.

*Conflict of interest*: None declared.

## Funding

This work was supported by the Innovative Medicines Initiative Joint Undertaking, under grant agreement No 115002 (eTOX) for O. Taboureau and No 115191 OPENPHACTS (S.K., J.K., T.I.O.) the resources of which are composed of financial contribution from the European Union's Seventh Framework Programme (FP7/2007-2013) and EFPIA companies in kind contribution.

## Supplementary Material

Supplementary Data
