# Supplementary material for: ChemProt-3.0: a global chemical biology diseases mapping
Source: Database (Oxford). 2016 Feb 14;2016:bav123. doi: 10.1093/database/bav123 (PMC4752971; doi:10.1093/database/bav123)
Supplement: Supplementary Data [file supp_2016_bav123_index.html]

ChemProt-3.0: a global chemical biology diseases mapping — Supplementary Data 

# ChemProt-3.0: a global chemical biology diseases mapping

## Supplementary Data

files

- Supplementary Data - pdf file
