# Supplementary material for: ChemProt-3.0: a global chemical biology diseases mapping
Source: Database (Oxford). 2016 Feb 14;2016:bav123. doi: 10.1093/database/bav123 (PMC4752971; doi:10.1093/database/bav123)
Supplement: Supplementary Data [file supp_bav123_Supplementary_revised-ChemProt_databaseJ.pdf]

Supplementary information: ChemProt-3.0: A global chemical-biology diseases mapping

Jens Kringelum, Sonny Kim Kjærulff, Tudor I. Oprea, Søren Brunak, Ole Lund and Olivier Taboureau.

Training of QSAR models

Dataset used for prediction

The ChemProt-3.0 dataset was used for generating QSAR models with the goal of creating accurate models capable of predicting interaction between novel chemical entities and proteins. The following activity types were used for training the models: IC_{50}, EC_{50}, Potency, AC_{50}, pIC_{50}, Log K_i, pK_i, pEC_{50}, K_d, K_i. The activity types not already converted were converted to –log10 values before used for prediction. If more than one value were present for a given chemical-protein pair the mean was used for model development. Proteins with less than 20 chemical interactions were excluded from the study. In total QSAR models for 2140 proteins were developed.

Generation of ensemble QSAR models

QSAR models were generated using a "one framework fits them all" approach to systematically perform QSAR models for all proteins included in ChemProt. To accommodate differences in the training datasets, a "wisdom of the crowd" framework using generic fingerprints and variable thresholds for classifying binders versus non-binder were adopted. QSAR models using classifiers were preferred over regression models, as classification tends to be more flexible and successful in prediction. To include a regression like scoring scheme the data was split in positives and negatives using 3 different –log10 values values; 4, 5 and 6, equivalent to 100 uM, 10 uM and 1 uM binding affinity. Classification models were trained on datasets split by each of the 3 thresholds.

The Naïve Bayes classifier was employed to relate chemical features (see below) to the measured activity class (positive or negative). It was found that, on a dataset of hERG binders/non binders (see below), that the Naïve Bayes classifier performed better or equal to other tested learning methods (i.e. Support Vector Machine (SVM) -Gaussian kernel, SVM -linear Kernel and Logistic Regression (LR) classifiers). Figure S1 outlines

the procedure used for training the QSAR models. One QSAR model will be trained for each combination of classification algorithms and chemical descriptor. In total 15 different QSAR models will be produced (5 descriptors types * 1 algorithms * 3 cutoffs for splitting data) for each protein in the dataset. The performance of each model was estimated in a 5-fold cross-validation scheme as outline in Figure S1, and used for weighting the prediction of each model when calculating the “wisdom of the crowd” score. Each dataset were balanced i.e. the same number of positive and negative (binders/non-binders) compounds were included in each dataset, by sampling the number of negative data points from the negative dataset corresponding to the number of positive data points present in the dataset. If not enough negative data were available random chemicals from ChemProt3.0 were included as negative data. Note that the final models used for prediction are trained on all data available.

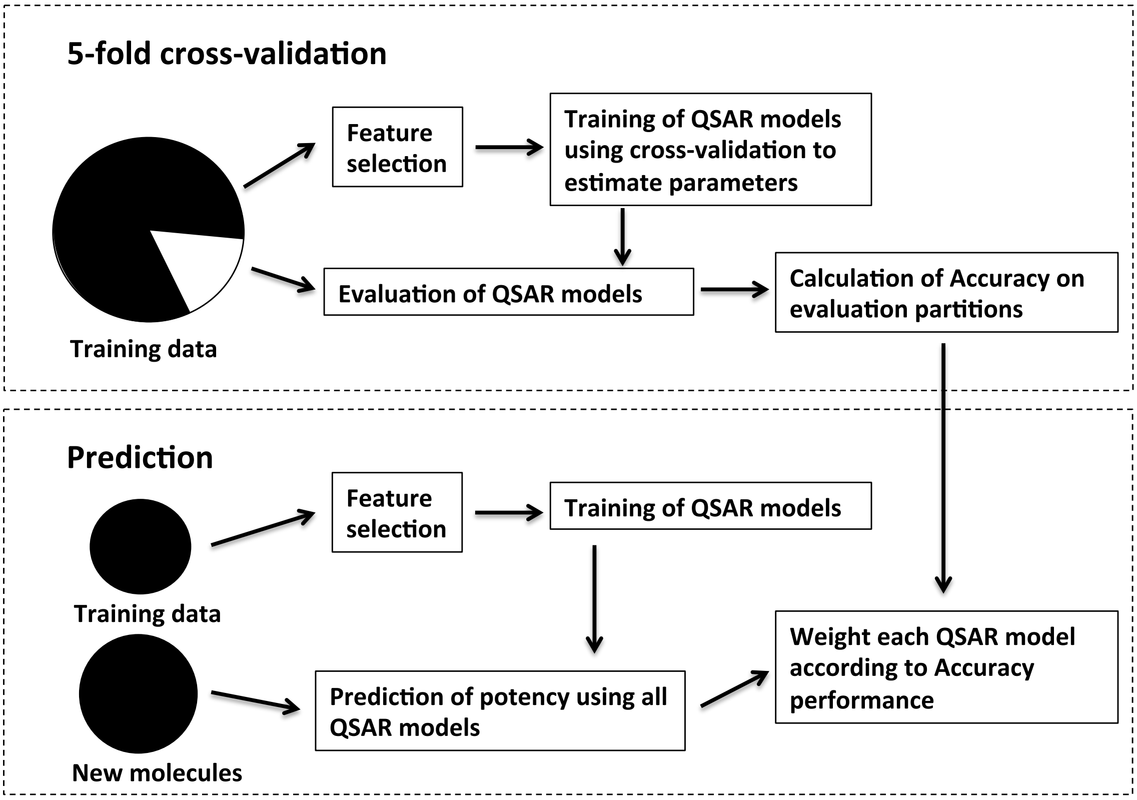

Figure S1. Outline of describe method. First, a 5-fold cross-validation scheme applied on the training data is used to determine the unbiased performance of each QSAR model. Next, all training data are used to generate QSAR models used for prediction of potency of new chemicals. Each prediction score (for each QSAR model) are weighted according to the model performance estimated in the 5-fold cross-validation scheme.

All models were trained using the scikit-learn software packages with a python wrapper (http://scikit-learn.org/stable/).

As the features space used to describe chemical structures is large compared to the available data, a features selection algorithm was employed to select a subset of features for model generation. A random forest approach, using the scikit-learn ExtraTreesClassifier with 100 trees, provided a consistence selection of features in a 5-fold cross-validation scheme on the hERG dataset. 100 trees were chosen to reduce running time as 15*6=90 feature selections have to be completed for each protein. Features were selected based on their average Gini-importance using the mean average Gini-importance as cutoff. The features selection are applied only to the training dataset in the cross-validation scheme, thus no bias is introduced towards descriptors general applicable to the dataset are introduced.

Performance measure

The accuracy score was used as measure of model performance for each of the 15 models generated for each dataset of interest (Figure S1). It converges to the Jaccard similarity score when the output is binary (classification) and gives the ratio between correctly classified instances and, correct + non-correct classification (total number of data points). Hence a complete random model will take the value of $ACC = 0.5$. As the dataset used are totally balanced the accuracy gives a reasonable estimation of the model performance and do not suffer from over-optimistic results biased against either negative or positive instances, as when applied to non-balanced datasets.

Predicting the potency of novel chemicals – "Wisdom of the crowd framework"

The scores were weighted based on model performance relative to the performance of a dummy model always outputting the average of training activity scores. As the dataset was balanced the model performance of the dummy model is always $ACC = 0.5$:

$$wm=ACCm-ACCdum=ACCm-0.5$$

The overall score was then calculated by weighting the predicted scores by the cross-validated performance as described in equation 2:

$$score=w1*S1+w2*S2+...+wm*Smw1+w2+...+wm$$

where w_m is the cross-validated performance (see Figure S1) and S_m is the predicted score (0 or 1) for each model:

Chemical Descriptors

As the chemical space are multi-dimensional and infinitely large, directly using chemical structures to build predictive models are not feasible. Instead descriptors are used that describe different features of the molecules, thereby transforming the structure into

features space. Multiple types of descriptors exist describing different molecular features. Here, the focus is on topological fingerprints (Daylight like fingerprints)[1] and Morgan fingerprints (also called circular fingerprints) [2].

Topological fingerprint will be called “daylight”. Morgan fingerprint will be called ECFP and FCFP for the atom and feature based version respectively to emphasize that the fingerprints utilize atom invariants connectivity information similar to those used for the well known ECFP family of fingerprints and feature-based invariants, similar to those used for the FCFP fingerprints.

It was chosen not to include pharmacophore fingerprints and 1D and 2D physical/chemical descriptors to keep the number of generated models at a reasonable level. Furthermore, the feature based Morgan fingerprint included is somewhat related to the 2D pharmacophore features as these describe the pharmacophore features around each atom in the chemical. All fingerprints were calculated using RDkit (www.rdkit.org) implemented in python. Figure S2 gives and overview of the chemical descriptors used in the presented work.

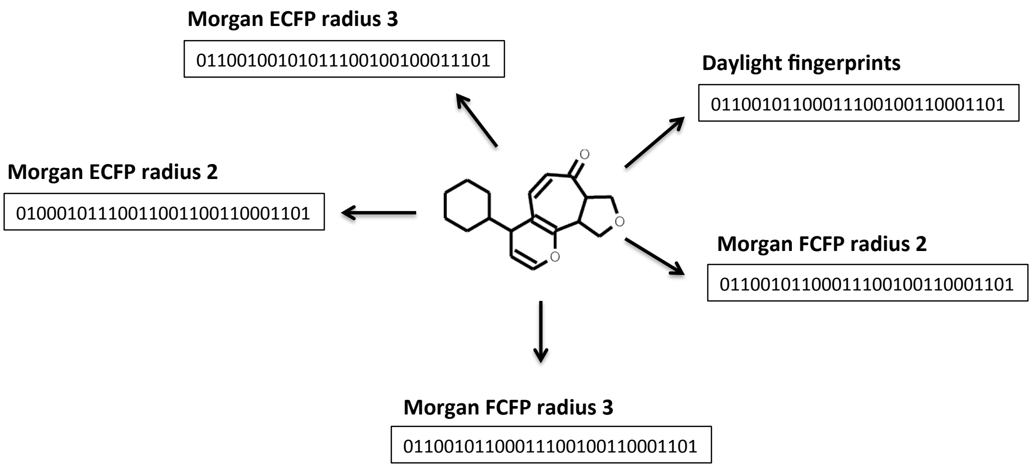

Figure S2. Chemical descriptors. In total 5 different chemical descriptors are used to generate QSAR models.

Prediction of the hERG-binders

To evaluate different settings such as the choice of prediction algorithm, number of bits in fingerprints and the added values of an ensemble approach, the hERG dataset obtains from [3] was used. A 5-fold cross-validation scheme was considered to estimate the

performance by splitting the dataset randomly into 5 different partitions and iteratively using 4 partitions for training and 1 for evaluation until all data points have been evaluated once (not to be confused with the cross-validation performed when training the ensemble models). The described ensemble approach was applied to each training partition i.e. training and evaluation dataset are kept totally separated during the training and evaluation. Thus no bias towards descriptors, classifier algorithms or training datapoints has been introduced, hence allowing selection of the best settings (classifier and number of bits) based on the cross-validated performance.

The IC50 (uM units) values used in the study were multiplied by -1 to reverse the scale and the cutoff -100, -10 and -1 uM were considered as the low, medium and high binder threshold respectively, except for single models – here -40 uM were used in agreement with the original study. Chemicals with an IC50 value below 40 uM were considered binders whereas chemicals with an affinity value above were considered non-binders. Some of the included performance measures require a binary classification, thus predicted value above 0.6 was regarded as binders for calculation of these performance measures.

Several different combinations of fingerprints types and lengths (number of bits) were tested as described in table S2. Using only a single fingerprint type reduces the performance of the model, however the FCFP and daylight fingerprints using 2048 bits still show reasonable performances. The length of the fingerprints (512, 1024 or 2048 bit) seems to influence performance slightly, whereas using a single fingerprint type consistently reduces the performance. However, inclusion of models trained using different classification algorithms (separately), boost performance (to a minor degree) with the non-linear algorithms performing the best (setting 16-19 in Table S2). Thus, choosing a single algorithm might be sufficient as long as it enables higher order correlations. Comparing the single models (setting 20-39) to the ensemble reveals that using an ensemble approach significantly enhanced prediction power. All ensemble models have improved prediction statistics compared to models only containing a single classification model (one single descriptor, one threshold and one classifier algorithm), even though the threshold used for splitting the training dataset into binders/non-binders for single models are the same used for the evaluation.

	Descriptors	Methods	Cutoffs	Roc	PCC	MCC	Sens	Spec	SCC
**2**	daylight_b1024<br>daylight_b2048<br>ECFP_b1024_r2<br>ECFP_b1024_r3<br>FCFP_b1024_r2<br>FCFP_b1024_r3<br>ECFP_b2048_r2	NaiveBayes<br>SVMlinear<br>LogisticRegressio<br>n<br>SVMGuassian	-100<br>-10<br>-1	0.849	0.602	0.481	0.634	0.841	0.607

	Descriptors	Methods	Cutoffs	Roc	PCC	MCC	Sens	Spec	SCC
	ECFP_b2048_r3<br>FCFP_b2048_r2<br>FCFP_b2048_r3								
**3**	daylight_b1024<br>daylight_b2048	NaiveBayes<br>SVMlinear<br>LogisticRegression<br>SVMGuassian	-100<br>-10<br>-1	0.820	0.560	0.486	0.723	0.778	0.546
**4**	ECFP_b1024_r2<br>ECFP_b1024_r3<br>FCFP_b1024_r2<br>FCFP_b1024_r3<br>ECFP_b2048_r2<br>ECFP_b2048_r3<br>FCFP_b2048_r2<br>FCFP_b2048_r3	NaiveBayes<br>SVMlinear<br>LogisticRegression<br>SVMGuassian	-100<br>-10<br>-1	0.844	0.586	0.497	0.660	0.835	0.606
**5**	daylight_b512<br>ECFP_b512_r2<br>ECFP_b512_r3<br>FCFP_b512_r2<br>FCFP_b512_r3	NaiveBayes<br>SVMlinear<br>LogisticRegression<br>SVMGuassian	-100<br>-10<br>-1	0.841	0.582	0.509	0.681	0.830	0.594
**6**	daylight_b1024<br>ECFP_b1024_r2<br>ECFP_b1024_r3<br>FCFP_b1024_r2<br>FCFP_b1024_r3	NaiveBayes<br>SVMlinear<br>LogisticRegression<br>SVMGuassian	-100<br>-10<br>-1	0.845	0.593	0.484	0.660	0.824	0.600
**7**	daylight_b2048<br>ECFP_b2048_r2<br>ECFP_b2048_r3<br>FCFP_b2048_r2<br>FCFP_b2048_r3	NaiveBayes<br>SVMlinear<br>LogisticRegression<br>SVMGuassian	-100<br>-10<br>-1	0.850	0.596	0.485	0.649	0.832	0.611
**8**	daylight_b2048	NaiveBayes<br>SVMlinear<br>LogisticRegression<br>SVMGuassian	-100<br>-10<br>-1	0.826	0.572	0.486	0.723	0.778	0.562
**9**	ECFP_b2048_r2	NaiveBayes<br>SVMlinear<br>LogisticRegression<br>SVMGuassian	-100<br>-10<br>-1	0.802	0.523	0.480	0.644	0.832	0.545
**10**	ECFP_b2048_r3	NaiveBayes<br>SVMlinear<br>LogisticRegression<br>SVMGuassian	-100<br>-10<br>-1	0.813	0.559	0.444	0.618	0.822	0.569
**11**	FCFP_b2048_r2	NaiveBayes<br>SVMlinear<br>LogisticRegression<br>SVMGuassian	-100<br>-10<br>-1	0.837	0.570	0.470	0.691	0.789	0.601
**12**	FCFP_b2048_r3	NaiveBayes<br>SVMlinear<br>LogisticRegression<br>SVMGuassian	-100<br>-10<br>-1	0.824	0.532	0.458	0.670	0.795	0.581
**13**	daylight_b2048<br>ECFP_b2048_r2<br>ECFP_b2048_r3<br>FCFP_b2048_r2<br>FCFP_b2048_r3	NaiveBayes<br>SVMlinear<br>LogisticRegression<br>SVMGuassian	-100	0.833	0.630	0.496	0.743	0.770	0.602
**14**	daylight_b2048<br>ECFP_b2048_r2<br>ECFP_b2048_r3<br>FCFP_b2048_r2<br>FCFP_b2048_r3	NaiveBayes<br>SVMlinear<br>LogisticRegression<br>SVMGuassian	-10	0.837	0.563	0.489	0.681	0.814	0.588
**15**	daylight_b2048<br>ECFP_b2048_r2<br>ECFP_b2048_r3<br>FCFP_b2048_r2	NaiveBayes<br>SVMlinear<br>LogisticRegression	-1	0.820	0.512	0.488	0.634	0.846	0.561

	Descriptors	Methods	Cutoffs	Roc	PCC	MCC	Sens	Spec	SCC
	FCFP_b2048_r3	SVMGuassian							
**16**	daylight_b2048<br>ECFP_b2048_r2<br>ECFP_b2048_r3<br>FCFP_b2048_r2<br>FCFP_b2048_r3	NaiveBayes	-100<br>-10<br>-1	0.843	0.574	0.513	0.634	0.865	0.608
**17**	daylight_b2048<br>ECFP_b2048_r2<br>ECFP_b2048_r3<br>FCFP_b2048_r2<br>FCFP_b2048_r3	SVMlinear	-100<br>-10<br>-1	0.838	0.568	0.500	0.670	0.830	0.595
**18**	daylight_b2048<br>ECFP_b2048_r2<br>ECFP_b2048_r3<br>FCFP_b2048_r2<br>FCFP_b2048_r3	LogisticRegressio<br>n	-100<br>-10<br>-1	0.838	0.592	0.468	0.660	0.811	0.606
**19**	daylight_b2048<br>ECFP_b2048_r2<br>ECFP_b2048_r3<br>FCFP_b2048_r2<br>FCFP_b2048_r3	SVMGuassian	-100<br>-10<br>-1	0.845	0.604	0.498	0.702	0.805	0.619
**20**	daylight_b2048	NaiveBayes	-40	0.687	0.398	0.355	0.717	0.657	0.364
**21**	daylight_b2048	SVMlinear	-40	0.685	0.431	0.352	0.733	0.638	0.414
**22**	daylight_b2048	LogisticRegressio<br>n	-40	0.713	0.472	0.404	0.770	0.657	0.472
**23**	daylight_b2048	SVMGuassian	-40	0.743	0.519	0.460	0.796	0.689	0.518
**24**	ECFP_b2048_r2	NaiveBayes	-40	0.727	0.452	0.445	0.670	0.784	0.463
**25**	ECFP_b2048_r2	SVMlinear	-40	0.706	0.439	0.392	0.723	0.689	0.431
**26**	ECFP_b2048_r2	LogisticRegressio<br>n	-40	0.674	0.356	0.330	0.707	0.641	0.353
**27**	ECFP_b2048_r2	SVMGuassian	-40	0.750	0.555	0.475	0.780	0.719	0.541
**28**	ECFP_b2048_r3	NaiveBayes	-40	0.752	0.502	0.494	0.707	0.797	0.503
**29**	ECFP_b2048_r3	SVMlinear	-40	0.703	0.431	0.388	0.712	0.695	0.425
**30**	ECFP_b2048_r3	LogisticRegressio<br>n	-40	0.720	0.440	0.419	0.749	0.692	0.457
**31**	ECFP_b2048_r3	SVMGuassian	-40	0.728	0.501	0.434	0.764	0.692	0.492
**32**	FCFP_b2048_r2	NaiveBayes	-40	0.770	0.514	0.518	0.775	0.765	0.526
**33**	FCFP_b2048_r2	SVMlinear	-40	0.744	0.492	0.462	0.812	0.676	0.509
**34**	FCFP_b2048_r2	LogisticRegressio<br>n	-40	0.737	0.477	0.449	0.806	0.668	0.480
**35**	FCFP_b2048_r2	SVMGuassian	-40	0.737	0.483	0.450	0.791	0.684	0.494
**36**	FCFP_b2048_r3	NaiveBayes	-40	0.753	0.505	0.487	0.743	0.762	0.510
**37**	FCFP_b2048_r3	SVMlinear	-40	0.720	0.445	0.419	0.749	0.692	0.433
**38**	FCFP_b2048_r3	LogisticRegressio<br>n	-40	0.721	0.422	0.421	0.759	0.684	0.436
**39**	FCFP_b2048_r3	SVMGuassian	-40	0.744	0.522	0.462	0.801	0.686	0.518
**40**	daylight_b1024<br>ECFP_b1024_r2<br>ECFP_b1024_r3<br>FCFP_b1024_r2<br>FCFP_b1024_r3	NaiveBayes	-100<br>-10<br>-1	0.827	0.562	0.488	0.649	0.835	0.579

Table S2. Cross-validated performance on the hERG binders. "daylight" denominates the topological fingerprint implemented in RDkit (essentially the same as daylight fps) and "_bXXXX" the number of bits used in the fingerprint. ECFP and FCFP is the Morgan circular atom and feature based fingerprints, the "_bXXXX" the number of bits used and "rX" the radius used in the circular fingerprint. Note that the performance values reported here are from the external cross-validation and not the cross-validation performed when training the ensemble of predictors described in Figure S1.

Comparison to the Similarity Ensemble Approach

The other prediction method implemented in ChemProt3.0 is the similarity ensemble approach (SEA) [4]. To compare the "new" QSAR implementation a dataset of 179 proteins of particular interest when investigating off-target effects were compiled (see Table S2). 143 of these had sufficient data available in ChemProt3.0 to train QSAR models and were used as the basis for comparing performance of the QSAR models to the SEA implementation. The dataset for the 143 proteins were spitted in 5-partition and a 5-fold cross-validation scheme were used to assess performances by using 4 partitions for training using the ensemble approach explained above and 1 partition for validation at a time. The partitions were spitted randomly.

For both, the ensemble QSAR model and SEA outputs float values spearman correlation coefficient (SCC) was used to compare performances. SCC is a parameter free coefficient (essential the PCC of ranked-values), which ensure a reasonable comparison even though the two methods output is on different scales. A SCC = 1 reflects perfect ranked-correlation between predicted and true values, 0 is random and -1 reflects an inverse correlation. Table S2 list the performances for the 143 proteins for both the ensemble QSAR predictive models and the SEA. Using a one-sided paired T-test and the null-hypothesis that the SCC_{QSAR} == SCC_{SEA} and the alternative hypothesis that SCC_{QSAR} > SCC_{SEA} the null-hypothesis could be rejected with a p-value of: 2.2e-16.

Uniprot id	Chemicals in dataset	Chemicals with aff < 100 *uM*	QSAR (MCC)	QSAR (SCC)	SEA (MCC)	SEA (SCC)
**O00408**	378	151	0.402451	0.50955	0.344713	0.43096
**O14920**	1478	659	0.321934	0.29005	-0.115163	-0.02482
**O15111**	740	586	0.339904	0.45846	0.045515	0.21119
**O43193**	346	185	0.445334	0.53197	0.029565	0.16587
**O75469**	401	183	0.134628	0.24651	0.486237	0.30118
**O76074**	1743	1220	0.369423	0.63437	0.173358	0.38443
**P00533**	6586	2971	0.208118	0.38331	0.068014	0.06649
**P00918**	5018	3130	0.306994	0.46368	0.233406	0.28322
**P02708**	173	82	0.322966	0.62251	0.443893	0.39995
**P03372**	4297	1910	0.063377	0.22351	0.058304	0.16055
**P04035**	399	327	0.544005	0.70464	0.463210	0.68764
**P04054**	715	411	0.202269	0.49507	0.012021	0.20465
**P04150**	3274	1358	0.222967	0.23143	-0.068802	0.02704
**P04626**	2835	1599	0.108386	0.16946	-0.108666	0.02532
**P06213**	1621	1333	0.219724	0.36826	0.151912	0.26055
**P06239**	3195	1750	0.228244	0.44590	-0.015748	0.09585
**P06241**	1160	981	0.052337	0.33172	0.064492	0.25434
**P06276**	1971	1076	0.356766	0.54489	0.406799	0.48597
**P06401**	2293	1018	0.486285	0.46309	0.260734	0.10618
**P07099**	223	152	0.461661	0.58652	-0.091246	0.46050
**P07550**	3543	2648	-0.209885	0.17583	-0.226525	0.12659
**P08172**	2577	1561	0.292175	0.47802	0.165528	0.27537
**P08173**	1475	918	0.247376	0.44268	0.266942	0.30187
**P08575**	446	203	0.630478	0.57311	0.513371	0.56767
**P08581**	2932	1561	0.434303	0.51074	0.103754	0.22970
**P08588**	2200	1174	0.365329	0.50309	-0.030187	0.18570
**P08908**	4490	3067	0.022687	0.29823	-0.104358	0.14712
**P08912**	1430	892	0.256736	0.44981	0.177523	0.30587

Uniprot id	Chemicals in dataset	Chemicals with aff < 100 *uM*	QSAR (MCC)	QSAR (SCC)	SEA (MCC)	SEA (SCC)
**P08913**	1294	751	0.242146	0.36677	0.107570	0.19707
**P09917**	2601	1099	0.401746	0.52336	0.406465	0.48313
**P10275**	3035	1392	0.198764	0.21697	0.030498	0.05820
**P10827**	574	282	0.110227	0.40767	0.291984	0.41157
**P10828**	6206	4347	0.123454	0.24354	0.038989	0.09952
**P11229**	3014	1802	0.289656	0.48636	0.180651	0.32294
**P11362**	2147	1276	0.502790	0.55929	-0.051391	0.06563
**P13945**	2051	943	0.356619	0.36245	0.113241	0.10493
**P14416**	6410	3965	0.251122	0.38451	0.130472	0.12944
**P15121**	947	348	0.446534	0.47176	0.289074	0.49439
**P16050**	5585	5332	-0.008573	0.05529	-0.034847	-0.04507
**P16499**	183	134	0.749976	0.72387	-0.056255	0.61923
**P17252**	1516	1032	0.256953	0.50621	0.063484	0.28475
**P18031**	3551	1557	0.227949	0.47487	0.335162	0.46771
**P18089**	864	452	0.328513	0.42126	0.092870	0.14488
**P18505**	392	318	0.563026	0.64087	0.521634	0.40597
**P18825**	969	532	0.084991	0.25204	0.092869	0.09428
**P20309**	2653	1853	0.360480	0.54712	0.260827	0.30244
**P21397**	1730	839	0.366523	0.58194	0.260285	0.30957
**P21452**	1305	875	0.246526	0.42711	0.214622	0.36293
**P21554**	4940	2876	0.329321	0.45572	0.147328	0.20142
**P21728**	1842	1357	0.088460	0.10742	0.064882	0.00330
**P21731**	1740	1081	0.341662	0.51589	0.390391	0.35465
**P21802**	306	189	0.222717	0.40674	0.144841	0.29963
**P21964**	28	20	0.918937	0.89737	0.825000	0.86275
**P22303**	4355	2044	0.265054	0.40367	0.298574	0.35173
**P22460**	723	417	0.544674	0.69627	0.321845	0.57291
**P23219**	2668	989	0.359692	0.45535	0.147578	0.31174
**P24385**	1027	820	0.436124	0.72183	0.156489	0.36897
**P24530**	1590	558	0.404737	0.41847	0.177662	0.22722
**P24557**	1582	1074	0.089215	0.27134	0.073330	0.20824
**P25021**	636	274	0.469123	0.47821	-0.204846	-0.26305
**P25025**	766	511	0.400073	0.61709	0.454360	0.54208
**P25100**	1794	1185	0.241764	0.41983	0.159083	0.31395
**P25101**	1935	879	0.330927	0.41738	0.222528	0.24149
**P25103**	3413	2102	0.296388	0.43976	0.177474	0.18279
**P25105**	1237	934	0.477019	0.60049	0.296159	0.31329
**P25929**	1583	1030	0.187363	0.48556	0.165193	0.38825
**P27338**	2023	1118	0.254571	0.40981	0.224359	0.18113
**P27361**	245	176	0.249885	0.45123	0.251427	0.46807
**P28222**	1499	847	0.335536	0.44544	-0.022992	0.03809
**P28223**	3680	2300	0.343386	0.46190	0.261365	0.38010
**P28335**	3674	2275	0.299806	0.43336	0.165062	0.30390
**P28482**	14750	14178	0.020024	0.06095	-0.025029	-0.01795
**P29274**	5283	3632	0.223482	0.37312	0.108488	0.02782
**P29275**	2963	1711	0.354750	0.45160	0.064439	-0.03379
**P29371**	745	451	0.474674	0.65848	-0.053988	0.27722
**P29474**	1220	567	0.148588	0.15770	0.353697	0.42580
**P29475**	1365	722	0.416119	0.54403	0.359333	0.51998
**P30411**	965	539	0.426062	0.46409	0.165504	0.26372
**P30518**	845	404	0.579142	0.53601	-0.005806	0.06917
**P30542**	4538	3120	0.243275	0.39789	0.132323	0.05837
**P30556**	2524	1958	0.251064	0.54028	0.327653	0.38584
**P30988**	68	66	0.252714	0.57826	-0.030303	-0.46397
**P32238**	1151	687	0.409047	0.54382	0.369426	0.49541
**P32239**	2212	1235	0.302717	0.41464	0.050701	0.10753
**P32245**	3679	2051	0.047348	0.26047	-0.177301	0.02098
**P32246**	927	379	0.532506	0.53562	0.122358	0.28447
**P33032**	924	423	0.054168	0.15490	0.110814	-0.05439
**P33765**	4561	2739	0.185105	0.29956	0.119639	0.22110
**P34969**	1466	961	0.305742	0.41693	0.027804	0.11401
**P34972**	5105	2738	0.253248	0.40855	0.210059	0.22529

Uniprot id	Chemicals in dataset	Chemicals with aff < 100 *uM*	QSAR (MCC)	QSAR (SCC)	SEA (MCC)	SEA (SCC)
**P35228**	1442	627	0.058933	0.09462	0.311075	0.33049
**P35348**	2207	1585	0.146633	0.36896	0.045436	0.13576
**P35354**	4199	1412	0.371908	0.41271	0.236921	0.22501
**P35367**	1807	1185	0.398152	0.56299	0.144463	0.28502
**P35368**	1998	1352	0.259582	0.44336	0.013839	0.20686
**P35372**	5842	3664	0.083636	0.30849	0.046352	0.19291
**P35408**	608	269	0.155132	0.30774	0.229855	0.25671
**P35462**	3776	2445	0.266060	0.41319	0.205926	0.26541
**P37231**	6072	2731	0.231398	0.39802	0.196543	0.29719
**P37288**	1144	766	0.419669	0.60248	0.104096	0.34641
**P41143**	5198	3272	0.108138	0.32721	0.149444	0.27068
**P41145**	5129	2909	0.290397	0.44092	0.267565	0.31076
**P41146**	1689	953	0.315774	0.43619	0.171341	0.26996
**P41595**	1787	1062	0.519684	0.54552	0.256756	0.35715
**P41597**	2093	914	0.466944	0.38929	0.133443	0.17903
**P41968**	1204	513	-0.020704	0.09257	0.030656	-0.05593
**P43116**	388	215	0.351130	0.47270	0.206393	0.16583
**P43403**	385	173	0.272454	0.63681	-0.001962	0.16855
**P43681**	1146	735	0.310845	0.43393	0.191214	0.19464
**P46098**	1013	729	0.228653	0.50812	0.065614	0.23347
**P46663**	912	554	0.484398	0.56007	0.218681	0.17882
**P47898**	612	516	0.078604	0.52912	-0.156730	0.29840
**P47901**	900	696	-0.088173	0.45766	-0.288398	-0.09535
**P48039**	1106	678	0.366601	0.45354	0.053589	0.12016
**P49146**	1044	740	0.040628	0.41250	-0.101508	0.21191
**P50052**	987	796	0.259462	0.45610	0.155775	0.05307
**P50406**	2873	1766	0.215509	0.36292	0.036120	0.03927
**P50416**	24	18	0.531085	0.63684	0.531085	0.32708
**P51679**	469	194	0.479826	0.43999	0.089631	0.33168
**P51681**	2857	1490	0.376459	0.45977	0.071800	0.09679
**P51955**	1109	961	0.178774	0.25443	0.099588	0.15846
**P54646**	166	123	0.413034	0.26751	0.279528	0.29725
**P83111**	84	24	0.419573	0.42666	0.133888	0.09205
**Q02763**	938	422	0.319639	0.43962	0.192030	0.34124
**Q08209**	63	48	0.625000	0.84545	0.199506	0.77218
**Q12809**	8181	4618	0.132471	0.25025	0.097760	0.12055
**Q13557**	907	806	0.175817	0.26655	0.154404	0.23731
**Q13639**	531	300	0.315139	0.46681	-0.046590	0.08110
**Q13936**	210	182	0.029951	0.54607	0.154091	0.45260
**Q14432**	1554	900	0.257560	0.44158	0.213886	0.21794
**Q14524**	514	284	0.568815	0.62064	0.524820	0.54123
**Q16539**	4970	2176	0.170355	0.29343	0.076182	0.11701
**Q8IW41**	641	568	0.097564	0.10988	0.185319	0.17787
**Q8NER1**	2503	1372	0.399808	0.49937	0.238143	0.36299
**Q92731**	3327	1280	0.363634	0.38105	0.295168	0.39229
**Q92847**	1660	825	0.509777	0.56865	0.323313	0.46232
**Q96EB6**	452	129	0.112290	0.31815	0.507464	0.46742
**Q96PF2**	519	510	-0.031213	-0.04255	-0.002228	-0.07087
**Q96RR4**	115	73	-0.160339	0.02679	-0.134806	0.21833
**Q9BZL6**	814	751	0.153985	0.18084	0.105034	0.18078
**Q9H2X6**	755	695	0.141024	0.35707	0.152371	0.32470
**Q9Y233**	923	603	0.528370	0.70836	0.076559	0.30252
**Q9Y5N1**	3474	2806	0.189611	0.39185	0.166854	0.24938
**All**	**2090**	**1268**	**0.288056**	**0.42782**	**0.151965**	**0.24415**

Table S2. Cross-validated performance of the selected off-target dataset. SCC is the spearman correlation coefficient; MCC is the Matthews correlation coefficient. MCC values were calculated by using a 100 uM threshold for true binders, 0.6 for QSAR models and 10^{-2} for the SEA model.

References

1. Ivanciuc O (2013) Chemical graphs, molecular matrices and topological indices in chemoinformatics and quantitative structure-activity relationships. Curr Comput Aided Drug Des 9: 153–163.

2. Rogers D et al. (2010) Extended-connectivity fingerprints. J Chem Inf Model 50: 742–754.

3. Li Q et al. (2008) hERG classification model based on acombination of support vector machine method and GRIND descriptors. Mol. Pharm. 5: 117-127.

4. Keiser MJ et al. (2007) Relating protein pharmacology by ligand chemistry. Nat. Biotechnol, 25: 197-206.
